# Effect of *Brassica rapa* L. Polysaccharide on Lewis Lung Cancer Mice by Inflammatory Regulation and Gut Microbiota Modulation

**DOI:** 10.3390/foods13223704

**Published:** 2024-11-20

**Authors:** Changhui Du, Yong Zhao, Fanglin Shen, He Qian

**Affiliations:** 1 School of Food Science and Technology, Jiangnan University, Wuxi 214122, China; 6210113015@stu.jiangnan.edu.cn; 2 Wuxi School of Medicine, Jiangnan University, Wuxi 214122, China; yongzhaojdfy@163.com; 3 School of Environmental Engineering, Wuxi University, Wuxi 214105, China; fanglinshen@cwxu.edu.cn

**Keywords:** *Brassica rapa* L. polysaccharide, lung cancer, inflammation, gut microbiota, SCFAs

## Abstract

Lung cancer is the leading cause of cancer-related fatalities globally, related to inflammatory and gut microbiota imbalance. *Brassica rapa* L. polysaccharide (BP) is a functional compound, which is utilized by the gut microbiota to regulate immunity and metabolism. However, the effect of BP on lung cancer and whether it affects the “gut–lung” axis remains unclear. This study explored the intervention of BP in Lewis lung cancer (LLC) mice and its effect on the gut microbiota. The results revealed that BP reduced tumor weight and downregulated the expression of Ki67 protein. Additionally, BP reduced the content of inflammatory factors and growth factors, promoting tumor cell apoptosis and inhibiting the growth of LLC. The intervention of BP suppressed intestinal inflammation, preserved intestinal barrier integrity, and augmented the level of beneficial microbiota, such as *Blautia* and *Bifidobacterium*. Furthermore, BP significantly increased the production of short-chain fatty acids (SCFAs), particularly butyrate and propionate. A correlation analysis showed significant correlations among the gut microbiota, SCFAs, inflammatory factors, and tight junction proteins. A functional analysis indicated that BP promoted amino acid metabolism and fatty acid metabolism. These findings suggested that BP had the potential to act as prebiotics to prevent disease and improve lung cancer progression by regulating the gut microbiota.

## 1. Introduction

Non-small cell lung cancer (NSCLC) is the most histological subtype of lung cancer, constituting around 80% of all the cases, significantly impacting human health and serving as a major cause of cancer-related mortality [1,2]. In clinical practice, surgery, radiotherapy, and chemotherapy are used to treat lung cancer; however, it is prone to develop drug resistance and have strong side effects. Recent research suggests that dysbiosis may modulate immune function and alter the effectiveness of tumor treatment [3]; there has been a notable increase in interest in cancer immunotherapy driven by the gut microbiota. The gut microbiota of NSCLC patients differed significantly from that of healthy people according to research on animal models and clinical trials [4,5]. Specifically, numerous studies indicated that the number of beneficial bacteria and microbial diversity decreases in the intestines of late-stage lung cancer patients with respect to the composition and diversity changes in the gut microbiota of advanced lung cancer patients [6]. The reduced abundance of bacteria such as *Actinobacteria*, *Bifidobacterium*, *Bacillus*, and *Clostridium butyricum* may serve as markers for the progression of lung cancer [7,8,9]. Moreover, the effect of the microbiota on treatment is extensive and includes the regulation of the efficacy and toxicity of chemotherapy and immunotherapeutic agents through metabolic and immune-mediated mechanisms [10]. Therefore, new response prediction biomarkers and methods for controlling the gut microbiota based on the “gut–lung axis” have been suggested for the treatment of lung cancer patients.

Tibetan turnip (*Brassica rapa* L.) is a medicinal and edible plant growing on the Tibetan plateau. According to Tibetan medicine, turnip is known for its sweet taste, warm nature, and its ability to clear heat, detoxify, nourish, and increase oxygenation. Polysaccharides are one of the important bioactive components in turnip, and research has indicated their potential functions, including anti-fatigue, anti-hypoxia, anti-inflammatory, and immune-modulatory effects [11,12,13]. Turnip considerably reduced the development of Lewis lung cancer cells in mice models of lung cancer and raised the amounts of B cells, CD4 T cells, CD8 T cells, and activated CD8 T cells in the spleens [14]. Liu et al. [15] discovered that *Brassica rapa* L. polysaccharides (BPs) exhibit prebiotic functions by increasing beneficial bacteria in both human and mouse intestines, producing short-chain fatty acids, and promoting amino acid metabolism and carbohydrate metabolism. More and more studies have shown that disruptions in the gut microbiota contribute to the development of diseases, and polysaccharides can be fermented by the gut microbiota in the colon to improve these conditions [16,17]. However, there is currently no evidence to reveal the impact of polysaccharides on the advancement of lung cancer from the perspective of the gut microbiota, and further research is necessary on its systematic, crucial target points and in-depth mechanisms of action.

The objectives of this study were as follows: evaluating the intervention effects of BP on lung cancer-bearing mice by assessing tumor weight, organ index, and cytokine levels, with a particular focus on its impact on the gut microbiota and the underlying regulatory mechanisms, thus providing a scientific foundation for the in-depth development of the dietary, nutritional, and medicinal value of polysaccharides.

## 2. Materials and Methods

### 2.1. Preparation of Brassica rapa L. Polysaccharides

*Brassica rapa* L. was provided by a farmland in Yushu, Qinghai province, China. The polysaccharides of *Brassica rapa* L. were prepared with reference to a previous study [11]. First, the crude polysaccharides were collected from the defatted material by ultrasonic-assisted extraction and then deproteinized by Sevag’s method. To further purify BP, stepwise ethanol precipitation was employed. The resulting precipitates were subsequently dissolved and dialyzed (7000 Da cut-off) for 48 h against distilled water. The BP obtained after lyophilization was used in the subsequent experiments; the purity of BP was 80.09% ± 3.74% with a relative molecular mass of 70.4 kDa [11]. The analytical grade reagents utilized were supplied by Sinopharm Chemical Reagent Co., Ltd. (Shanghai, China).

### 2.2. Animal Experiment

C57BL/6 mice (male, 6 weeks, 18–22 g, specific-pathogen-free) were purchased from HuaChuang Sino (Taizhou, Jiangsu, China). The animal procedures were implemented in conformity with the Guidelines for Care and Use of Laboratory Animals of Jiangnan University and the animal protocols were authorized by the Animal Ethics Committee of Jiangnan University (JN.No20230830c0641230[348]). The mice were housed in a 12 h dark/light cycle at 24 ± 2 °C and 60% relative humidity.

The mice were randomly separated into 4 groups (*n* = 8) following a week of dietary acclimatization, including control group (Con), model (Mod), low dose of BP (BPL, 100 mg/kg), and high dose of BP (BPH, 200 mg/kg) [15]. All the mice were provided with a standard diet and water. Lewis lung carcinoma (LLC) cells were procured from the Cell Bank of the Chinese Academy of Sciences (Shanghai, China) and then cultivated in DMEM supplemented with 10% FBS in an incubator with saturated humidity and 5% (*v*/*v*) CO_2_ at 37 °C and passaged every 2–3 days. Except for the control group, 4 × 10^5^ LLC cells were injected subcutaneously into the right flank of the mice [18]. The Con and Mod groups were given physiological saline by gavage, and corresponding doses of polysaccharides were given BPL and BPH simultaneously with LLC on day 0. Positron emission tomography/computed tomography (PET/CT) was used to visualize the tumor. After receiving gastric intubation for 21 days, all the mice were euthanized, and blood and tissue samples of mice were taken and stored at −80 °C until analysis.

### 2.3. Biochemical Indexes Assay

The collected blood was centrifuged at 3000 rpm for 15 min at 4 °C to separate the serum from the blood cells. The content of various cytokines, including interleukin (IL)-1β, IL-6, IL-10, tumor necrosis factor-alpha (TNF-α), C-reactive protein (CRP), interferon-γ (IFN-γ), transforming growth factor-β (TGF-β), vascular endothelial growth factor (VEGF), and platelet-derived growth factor (PDGF-BB) in the serum of the mice, was measured by Enzyme-linked immunosorbent assay (ELISA) kits in accordance with the manufacturers protocol from Shanghai Kexing Trading Co., Ltd., Shanghai, China.

### 2.4. Tumor Apoptosis Detection

The fixed tumor tissue was collected, paraffin-embedded, and cut into 4 μm slices. Terminal transferase, deoxyuridine triphosphate, and equilibration buffer were added and mixed according to the Tunel kit instructions. DAPI stain was added and incubated for 10 min in the dark, washed with PBS, and shaken dry, and the sections were sealed with anti-fluorescence quenching sealer. Finally, the sections were observed under a fluorescence microscope (Olympus, Tokyo, Japan).

### 2.5. Hematoxylin and Eosin (H&E) and Immunocytochemistry (IHC) Staining

The colon tissue and tumor tissue were embedded in paraffin after being treated with 4% paraformaldehyde. Subsequently, the wax was dried on slides after being sectioned into 5 μm slices. The samples were dehydrated using graded ethanol, and the wax segments were dewaxed using xylene. Then, the slices were stained with H&E and oil red O staining, and finally subjected to an inverted microscope.

The paraffin slices of colon tissue and tumor tissue were dewaxed in water and then antigen was extracted using sodium citrate buffer. The sections were co-incubated with the primary antibody after 30 min of blocking with a 3% bovine serum albumin solution. Subsequently, the sections were incubated for 50 min after the addition of a mouse/rabbit universal secondary antibody. Diaminobenzidine (DAB) chromogen was utilized for visualization, followed by counterstaining with hematoxylin. Finally, immunohistochemical images were obtained using an optical microscope and processed by the Image-Pro Plus 6.0 software (National Institutes of Health, Bethesda, MD, USA).

### 2.6. RNA Extraction and Quantitative Real-Time PCR (qRT-PCR)

Total RNA was extracted from the mice colon by the Tissue Total RNA Isolation Kit (Vazyme Biotech Co., Ltd., Nanjing, China). The cDNA reverse transcription kit was used to reverse transcribe the resulting RNA into cDNA. The SYBR qPCR Master Mix kit was used to perform q-PCR and detect the relative expression content of the target gene. β-actin was used as the reference gene, and the primers used are shown in Table 1.

### 2.7. 16S rRNA Gene Sequencing Analysis

Fresh fecal samples were collected from each mouse before euthanasia to analyze the gut microbiota composition. DNA was extracted by the fecal Fast-DNA Spin Kit (MP Biomedical, CA, USA), and agarose gel electrophoresis was used to evaluate the DNA quality and purity. Then, primer-based polymerase chain reaction (PCR) amplification of 16S rRNA (V3-V4 region) was carried out at 98 °C for 1 min, then 30 cycles of denaturation for 10 s at 98 °C, annealing for 30 s at 50 °C, 72 °C for 30 s, and lastly extending for 5 min at 72 °C. The PCR products were purified using the Qiagen Gel Extraction Kit (Qiagen, Hilden, Germany), and the qualified sequencing library was sequenced by TruSeq^®^ DNA PCR-Free Sample Preparation Kit (Illumina, San Diego, CA, USA).

The QIIME software (v1.9.1, http://qiime.org/scripts/split_libraries_fastq.html, accessed on 7 October 2024) was used to search for systematic phylogenetic relationships, and sequences that showed 97% identity were allocated to the same operational taxonomic units (OTUs). The results were then entered into the Greengenes database. The Kyoto Encyclopedia of Genes and Genomes (KEGG) database was utilized to conduct the gut microbial genomic function study using the Tax4Fun2 software (v1.1.5, https://sourceforge.net/projects/tax4fun2/, accessed on 7 October 2024).

### 2.8. Short-Chain Fatty Acids (SCFAs) Determination

According to the method described by Liu et al. [19], the mice colon contents were dissolved in 1 mL of dimethyl carbonate, 0.1 mL of KHSO_4_ saturated solution, and 0.1 mL of 2-hydroxybutyric acid internal standard solution (0.1 mg/mL). The mixture was violently shaken for 10 min to ensure thorough mixing, and then centrifuged at 4000× *g* for 10 min to obtain the dimethyl carbonate phase to prepare the standard solution for the pretreatment of SCFAs in the range of 0.05–5.0 mg/mL. Finally, the amounts of fecal SCFAs were examined using the GC-MS.

### 2.9. Statistical Analysis

The information was given as mean ± standard deviation (x ± SD). A one-way analysis of variance (ANOVA) with Dunnett’s multiple comparisons test was applied to analyze the statistical differences between different groups. A *p*-value less than 0.05 was considered statistically significant.

## 3. Results

### 3.1. Influence on Tumor Progression of LLC Mice

#### 3.1.1. Influence on Body Weight and Tumor Weight

**Figure 1:**
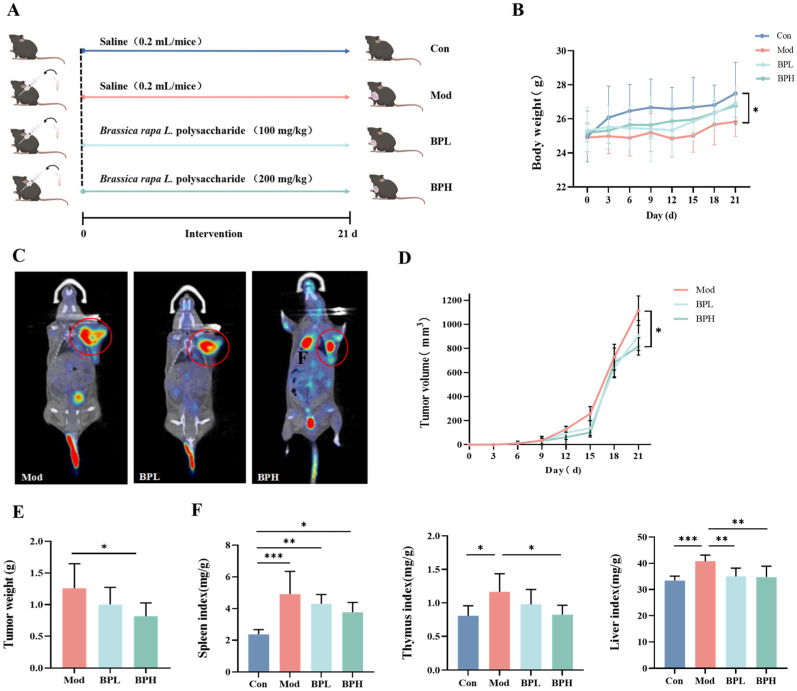
Effect of *Brassica rapa* L. polysaccharide (BP) on body weight, tumor, and organ index in lung cancer mice. (**A**) Scheme of animal experiments. (**B**) Body weight. (**C**) Positron emission tomography/computed tomography (PET-CT) imaging of lung cancer mice (**D**) Tumor volume. (**E**) Tumor weight. (**F**) Organ index. * *p* < 0.05, ** *p* < 0.01, *** *p* < 0.001.

During the intervention course of 21 d time (Figure 1A), visible subcutaneous tumors were observed in the mice on days 7–10, indicating successful modeling. The body weight of the mice inoculated with tumors increased slowly (Figure 1B); in the Mod group, it was significantly lower than that of mice in the Con group (*p* < 0.05).

Tumor growth was observed over time, as shown in Figure 1C,D; the tumor volume reached 1113.93 mm^3^ at day 21, and the BPH group was 816.56 mm^3^, which was significantly lower than the Mod group (*p* < 0.05). The tumor weight in the mice fed with BP was markedly reduced at the end of the experiments compared with the model group (Figure 1E). Especially, a high dose of the BP (BPH vs. Mod, *p* < 0.05) was more effective than a low dose in inhibiting tumor growth.

#### 3.1.2. Influence on Organ Index

In addition, Figure 1F shows the organ index (the spleen, thymus, and liver) of the mice, respectively. The increase in organ coefficients suggests possible inflammatory changes in the organs, such as congestion, edema, and hypertrophy. The coefficients of the liver and immune organ (spleen and thymus) in the Mod group were both significantly higher compared to the Con group (*p* < 0.05). BP had a significant alleviative effect; particularly, high-dose BP markedly reduced the thymus index in tumor-bearing mice to a normal level (*p* < 0.05), indicating the role of BP intervention in protecting immune organs and regulating immune function.

#### 3.1.3. Influence on Histopathology of Tumors

**Figure 2:**
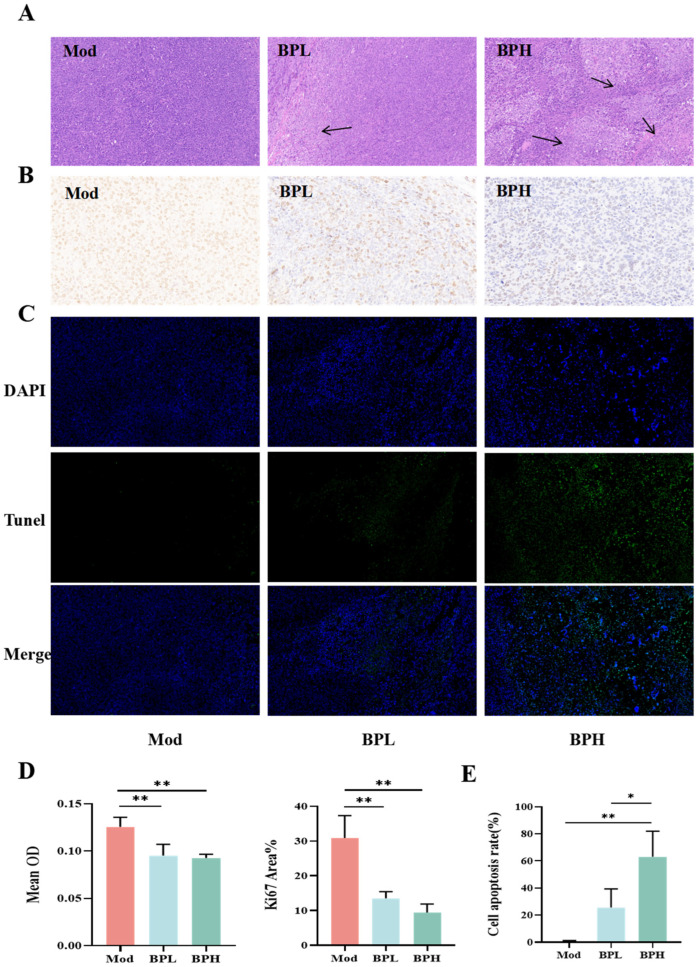
Effect of BP on histopathology in lung cancer mice. (**A**) Tumor tissue pathology (×100). (**B**) Immunohistochemistry of Ki67 (×400). (**C**) Immunofluorescence (×200). (**D**) The average OD value and the area of Ki67-positive cells. (**E**) The proportion of Tunel-positive cells. BPL: low dose of BP; BPH: high dose of BP. * *p* < 0.05; ** *p* < 0.01.

The HE staining of tumor tissue is shown in Figure 2A. The tumor cells in the Mod group were densely arranged with a relatively high degree of proliferation and deep staining. The tumor cells of the BPL group had a looser arrangement and lighter nuclear staining of tumor cells. The tumor cells in the BPH group showed nuclear division and disintegration and extensive necrosis.

Ki67 immunohistochemical staining was performed to clarify the effect of BP on tumor cell proliferation. As shown in Figure 2B, the Mod group showed a large number of Ki67-positive cells, indicating that the tumor cells were in a state of rapid proliferation. BP significantly reduced the average optical density value and the area percentage of positive cells compared with the model group (BPH vs. Mod, *p* < 0.01), and the area percentage of Ki67-positive cells was reduced from 30.86% to 9.42% (Figure 2C). The Tunel staining method can be used to observe the apoptosis of tumor cells. The broken DNA showed green fluorescence when apoptosis occurred, as shown in Figure 2D, and the number of apoptotic cells increased under the intervention of BP. Compared with the mice in the Mod group and BPL group, the proportion of Tunel-positive cells in the BPH group was significantly higher (*p* < 0.05). This finding suggested that BP inhibited tumor cell proliferation and promoted apoptosis.

### 3.2. Influence on Serum Cytokines of LLC Mice

**Figure 3:**
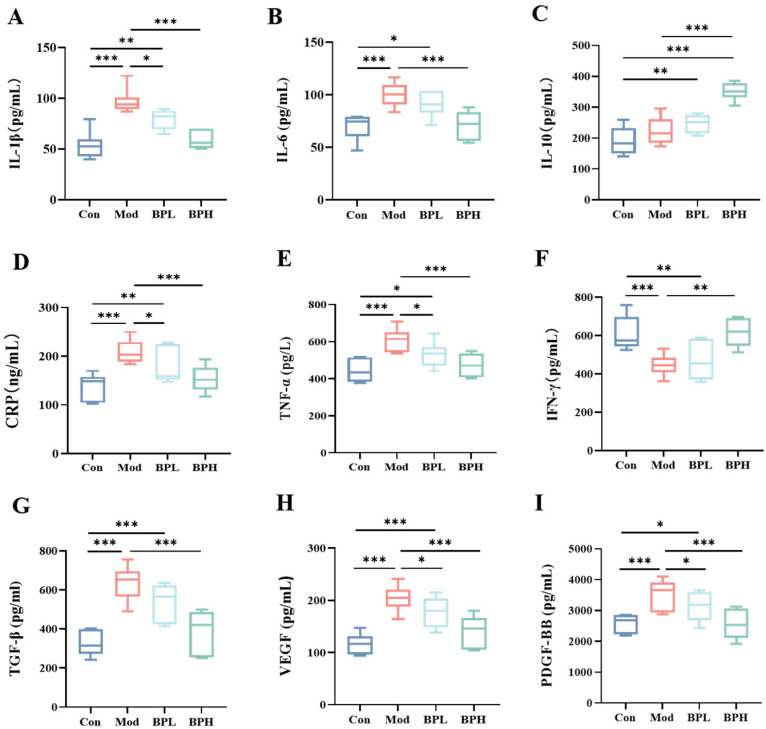
Effect of BP on serum cytokines in lung cancer mice. (**A**) IL-1β. (**B**) IL-6. (**C**) IL-10. (**D**) CRP. (**E**) TNF-α. (**F**) IFN-γ. (**G**) TGF-β. (**H**) VEGF. (**I**) PDGF-BB. * *p* < 0.05, ** *p* < 0.01, and *** *p* < 0.001.

The concentrations of inflammatory cytokines and growth factors in the serum of the mice from various treatment groups were measured using ELISA (Figure 3). After inoculation with LLC, pro-inflammatory cytokines IL-1β, IL-6, and TNF-α increased, showing significant differences between the Con and Mod groups (*p* < 0.001). Elevated levels of IL-1β, IL-6, and TNF-α have been demonstrated to facilitate the development of cancer [20]. The levels of IL-10 in the lung cancer mice were elevated, which may be due to the autoimmune defense generated by the tumor. The IL-10 levels of the BPH group increased from 226.35 pg/mL to 351.13 pg/mL (BPH vs. Mod, *p* < 0.001). The content of CRP in the Mod group increased from 136.57 ng/mL to 210.18 ng/mL (*p* < 0.001), while the intervention of BP significantly reduced. IFN-γ exhibited anticancer activity by inhibiting cell growth, cytotoxicity, and migration [21,22]. The levels of IFN-γ in the Mod group, BPL group, and BPH group were 445.73 pg/mL, 469.87 pg/mL, and 617.18 pg/mL; high-dose BP significantly increased the level of IFN-γ (*p* < 0.01).

Serum levels of TGF-β, VEGF, and PDGF-BB were measured to assess vascular growth and cancer progression [23]. The levels of TGF-β in the Mod group were elevated from 332.14 pg/mL to 631.87 pg/mL (*p* < 0.001), whereas the BPL and BPH group were restored to 548.0 pg/mL, 388.20 pg/mL (*p* > 0.05, *p* < 0.001). Compared to the Con group, mice in the Mod group had significantly higher levels of VEGF and PDGF-BB (*p* < 0.001), and BP was able to significantly reduce VEGF and PDGF-BB levels, inhibiting tumor cell migration. The administration of BP lowered the levels of inflammatory factors and growth factors, indicating that BP can enhance immune regulatory capacity and inhibit angiogenesis to suppress the progression of lung cancer.

### 3.3. Influence on Intestinal Barrier of LLC Mice

#### 3.3.1. Influence on Intestinal Inflammatory Factors

**Figure 4:**
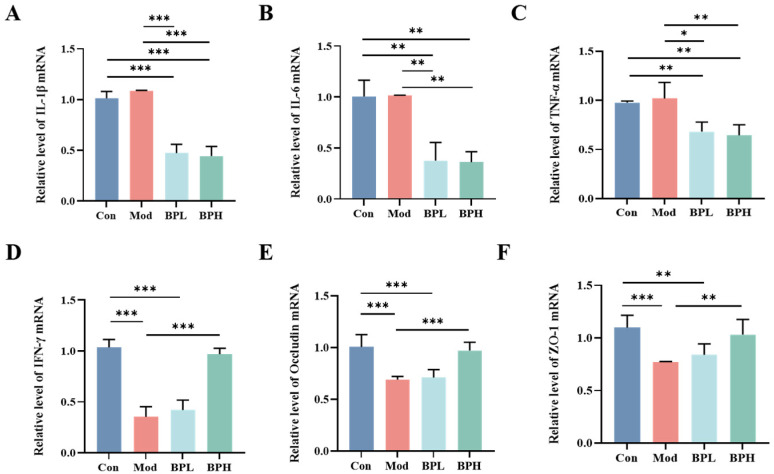
Effect of BP on inflammatory factors and tight junction protein-related genes in the intestine of lung cancer mice. (**A**) Relative level of IL-1β. (**B**) Relative level of IL-6. (**C**) Relative level of TNF-α. (**D**) Relative level of IFN-γ. (**E**) Relative level of Occludin. (**F**) Relative level of ZO-1. * *p* < 0.05, ** *p* < 0.01, and *** *p* < 0.001.

As shown in Figure 4, the mRNA relative expression of inflammatory factors IL-1β, IL-6, TNF-α, and IFN-γ in colon tissues was measured by qRT-PCR. The relative expression of IL-1β, IL-6, and TNF-α mRNA in the intestines of the Mod group did not change significantly compared to the Con group, and the relative expression of IFN-γ mRNA was significantly reduced (Mod vs. Con, *p* < 0.01). Compared with the Con and Mod groups, the relative expressions of IL-1β, IL-6, and TNF-α mRNA in the BP intervention groups were significantly decreased (*p* < 0.05), and the relative expression of the IFN-γ mRNA was significantly increased (*p* < 0.001). Pro-inflammatory factors and IFN-γ were associated with the permeability of the intestinal epithelial tight junction barrier [24,25]. Tight junction proteins such as Occludin and Zonula occludens-1 (ZO-1) play an essential role in maintaining intestinal mucosal integrity, and changes in expression are widely utilized as indicators of intestinal disruption [26]. The relative mRNA expression of ZO-1 and Occludin was significantly reduced in the colon tissue of the LLC mice compared to the normal mice (*p* < 0.001). Under the intervention of BP, the high dose of BP significantly increased the relative mRNA expression of Occludin and ZO-1 (*p* < 0.01), which restored them to the normal level. This suggested that BP could reduce the expression of pro-inflammatory factors, reduce intestinal damage, and maintain intestinal barrier homeostasis.

#### 3.3.2. Influence on Intestinal Histopathology

**Figure 5:**
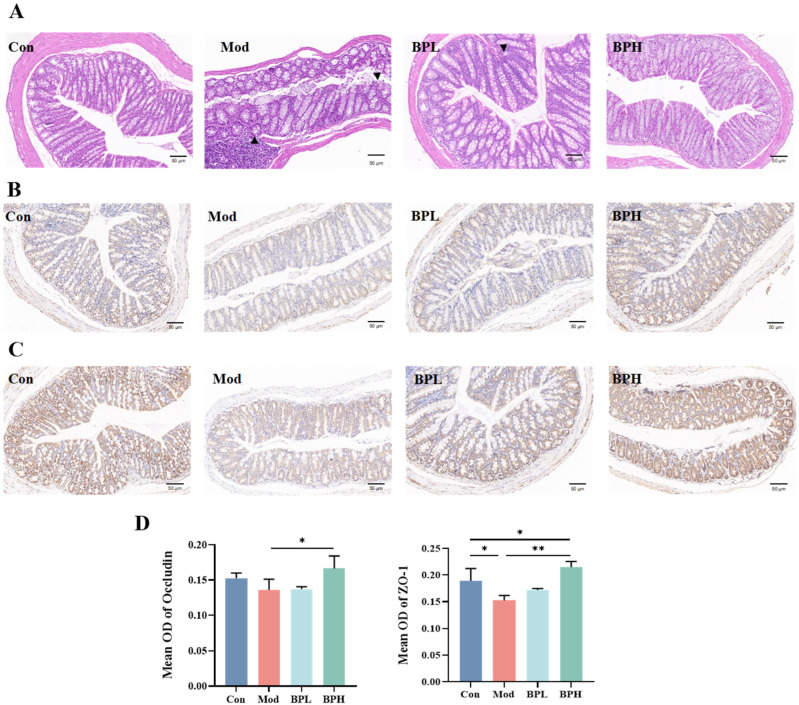
Effect of BP on colon tissues and tight junction proteins of lung cancer mice. (**A**) HE staining of colon tissues (×200). (**B**) IHC staining of Occludin (×200). (**C**) IHC staining of ZO-1 (×200). (**D**) Mean OD of Occludin and ZO-1. * *p* < 0.05, ** *p* < 0.01.

To investigate the effect of BP on the intestines, H&E staining was evaluated to analyze the histological improvement of colon tissues following BP intervention (Figure 5A). The colonic mucosal epithelium of the control group mice remained intact, but the colonic mucosa of the Mod group mice was incomplete, characterized by sparse and disordered cell arrangement, along with a significant infiltration of inflammatory cells. The low- and high-dose BP intervention groups exhibited better maintenance of colonic epithelial tissue integrity, with reduced inflammatory cell infiltration and an indication of increased goblet cell numbers.

#### 3.3.3. Influence on Intestinal Tight Junction Proteins

As shown in the immunohistochemical images in Figure 5B,C, the protein levels of Occludin and ZO-1 in the colon tissues of the Mod group mice exhibited significant reduction compared to the Con group. Figure 5D shows that the BP intervention elevated the average optical density values of Occludin and ZO-1, with the high-dose group having the better effect (BPH vs. Mod, *p* < 0.05). The collective findings indicated that BP inhibited this protein expression change in the colon tissue, and preserved the normal physiological function of the intestinal barrier, thereby alleviating the damage to the intestines caused by LLC tumor-bearing in mice.

### 3.4. Influence on Gut Microbial Composition of LLC Mice

#### 3.4.1. Influence on Alpha and Beta Diversity

**Figure 6:**
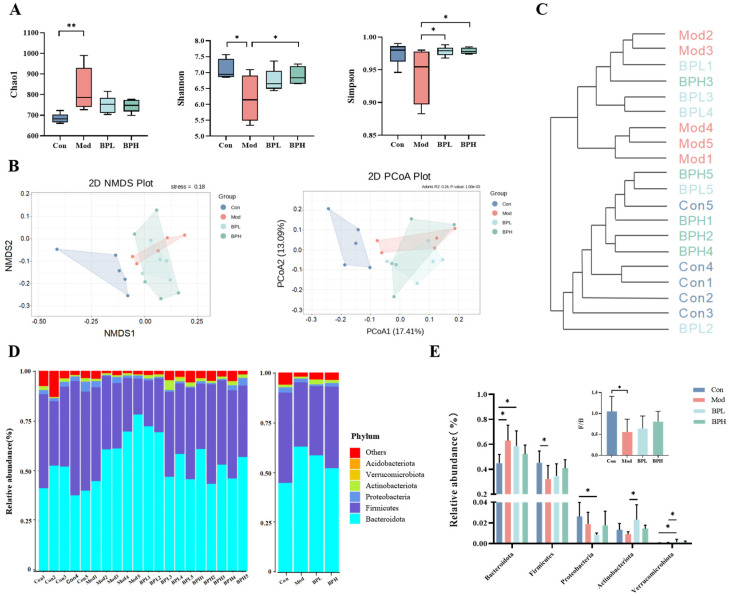
Effect of BP on microbiota in lung cancer mice. (**A**) α-diversity. (**B**) Beta-diversity. (**C**) Cluster tree. (**D**,**E**) Relative abundance of microbiota at phylum level. * *p* < 0.05, ** *p* < 0.01.

Next, we employed 16S rRNA sequence analysis to investigate the influence of BP on the gut microbiota structure and composition in mice with lung cancer. In Figure 6A, the Chao1 index of the Mod group was considerably higher than that of the Con group (*p* < 0.01). However, the intake of BP significantly increased the Shannon and Simpson indices, indicating that BP enhanced the diversity of the intestinal microbiota in mice. The results of beta diversity and cluster analysis (Figure 6B,C) showed significant variations in community composition between the Con group and the groups of Mod, BPL, and BPH, suggesting that BP affected the composition of intestinal microbiota in mice.

#### 3.4.2. Influence on Gut Microbiota Composition

At the phylum level, the dominant bacteria in each group were primarily Bacteroidota, Firmicutes, Proteobacteria, Actinobacteriota, and Verrucomicrobia (Figure 6D). After tumor inoculation, the relative abundance of Bacteroidota significantly increased and Firmicutes significantly decreased compared with the Con group (Mod vs. Con, *p* < 0.05). The ratio of Firmicutes to Bacteroidetes (F/B) is a crucial indicator of microbial imbalance and is used to evaluate the degree of inflammation in diseases [27]. Figure 6E shows that the F/B ratio of the Mod group was considerably decreased (*p* < 0.05), whereas the BP intervention groups performed an increase in the F/B ratio.

**Figure 7:**
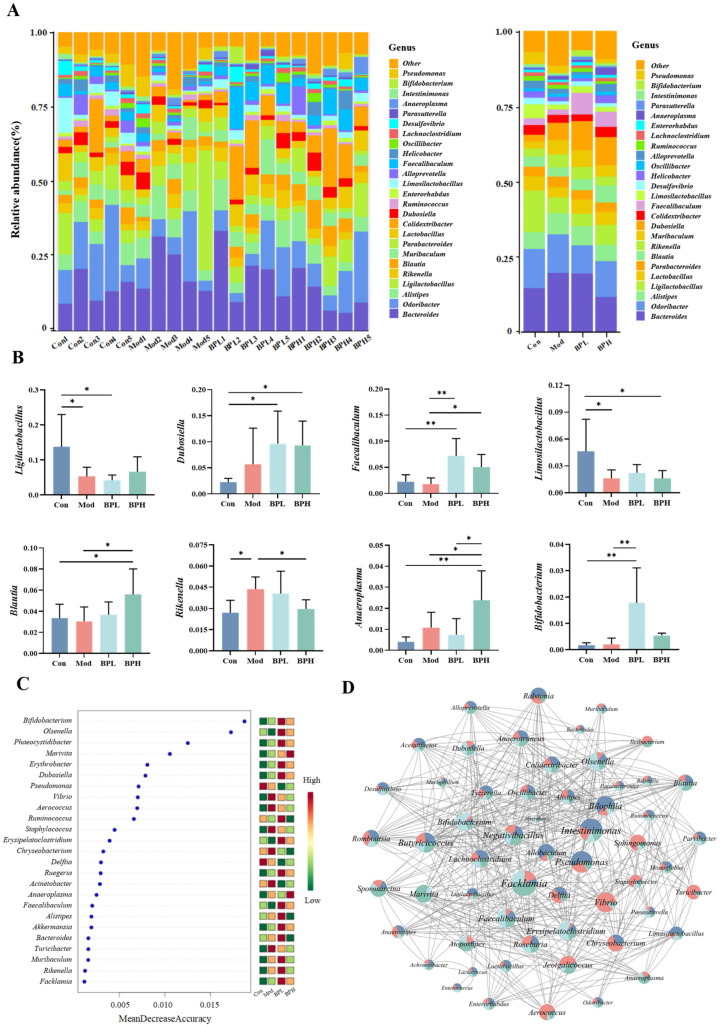
Effect of BP on differential microbiota in lung cancer mice. (**A**) The relative abundance of gut microbiota; (**B**) analysis of key microorganisms. (**C**) Random forest analysis. (**D**) Microbial co-occurrence network. * *p* < 0.05, ** *p* < 0.01.

The composition of the intestinal microbiota at the genus-level analysis was shown in Figure 7A, essentially composed of *Bacteroides*, *Odoribacter*, *Alistipes*, *Ligilactobacillus*, *Lactobacillus*, *Parabacteroides*, *Blautia*, *Rikenella*, *Dubosiella*, and *Faecalibaculum*. The Mod group showed a substantial drop in the relative abundance of *Ligilactobacillus* and *Limosilactobacillus* while the relative abundance of *Rikenella* increased compared to the Con group (*p* < 0.05). Nevertheless, this trend was significantly reversed after the BP intervention. Additionally, the low-dose group exhibited a noteworthy rise in the content of *Faecalibaculum* and *Bifidobacterium* (*p* < 0.01), and the high-dose group showed a substantial increase in the content of *Dubosiella*, *Faecalibaculum*, *Blautia*, and *Anaeroplasma* (*p* < 0.05).

**Figure 8:**
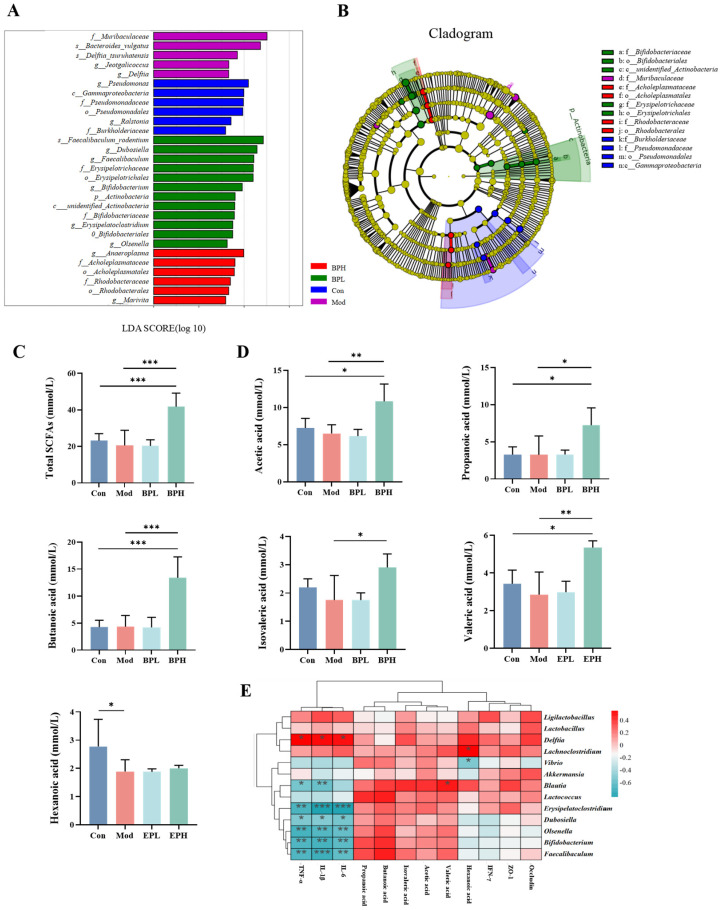
Effect of BP on gut microbiota and SCFAs in lung cancer mice. (**A**) LDA score. (**B**) LEfSe of microbiota. (**C**) Total SCFAs. (**D**) SCFA concentration. (**E**) Correlation analysis of SCFSs, inflammatory factors, tight junction protein, and microbiota. * *p* < 0.05, ** *p* < 0.01, and *** *p* < 0.001.

In Figure 7B,C, a random forest analysis identified the top 25 differential abundant microbial genera. The Mod group exhibited a positive correlation with *Turicibacter*, *Vibrio*, *Staphylococcus*, and *Delftia*, while it displayed a negative correlation with the *Faecalibaculum*, *Erysipelotrichaceae*, and *Olsenella*. Some probiotics, such as *Rikenella*, *Akkermansia*, and *Bifidobacterium*, were positively associated with the BP intervention groups. To further explore the effects of BP on the gut microbiota, a co-abundance network of genus-level microorganisms with higher relative abundance was constructed (Figure 7D). The results indicated that the main related bacteria with strong correlations were *Facklamia*, *Negativibacillus*, *Lachnoclostridium*, *Ligilactobacillus*, *Faecalibaculum*, and *Bifidobacterium*, indicating the mutual influence and interaction among microbiota. Similarly, the LDA analysis in Figure 8A,B demonstrated that the characteristic microorganisms for the Mod group were *Muribaculaceae*, *Jeotgalicoccus*, and *Delftia*, while the characteristic microorganisms for the BP intervention were *Dubosiella*, *Faecalibaculum*, *Bifidobacterium*, and *Olsenella*.

### 3.5. Influence on SCFAs of LLC Mice

Figure 8C,D shows that the concentration of SCFAs in the colon contents of the BPH group was 42 mM, which was higher than the Con and Mod groups (*p* < 0.001). After being metabolized by the gut microbiota of the mice, copious amounts of acetic acid and propionic acid were produced. It is reported that butanoic acid and valeric acid can improve the antitumor activity of immune cells through metabolism and epigenetic reprogramming [28]. High-dose BP increased the butanoic acid and valeric acid concentrations from 4 mM to 13 mM, and 3 mM to 5 mM, respectively, in lung cancer mice (*p* < 0.01).

A correlation heatmap (Figure 8E) was utilized to identify potential interactions among the gut microbiota, SCFAs, and the relative mRNA levels of intestinal inflammatory factors and tight junction proteins. The intestinal inflammatory factors IL-1β, IL-6, and TNF-α showed a significant negative correlation with *Blautia*, *Erysipelatoclostridium*, *Dubosiella*, *Olsenella*, *Bifidobacterium*, and *Faecalibaculum* (*p* < 0.05), while it showed a significant positive correlation with *Delftia* (*p* < 0.05). The relative mRNA levels of Occludin and ZO-1 were positively correlated with *Ligilactobacillus*, *Akkermansia*, *Lachnoclostridium*, and *Blautia*, suggesting that the gut microbiota could contribute to the maintenance of intestinal barrier function integrity by promoting the expression of tight junction proteins. *Lachnoclostridium*, *Blautia*, *Lactobacillus*, *Olsenella*, *Bifidobacterium*, and *Faecalibaculum* showed significant positive correlations with SCFAs, suggesting that the gut microbiota could utilize BP to increase the contents of SCFAs.

### 3.6. Influence on Microbial Metabolic Function of LLC Mice

**Figure 9:**
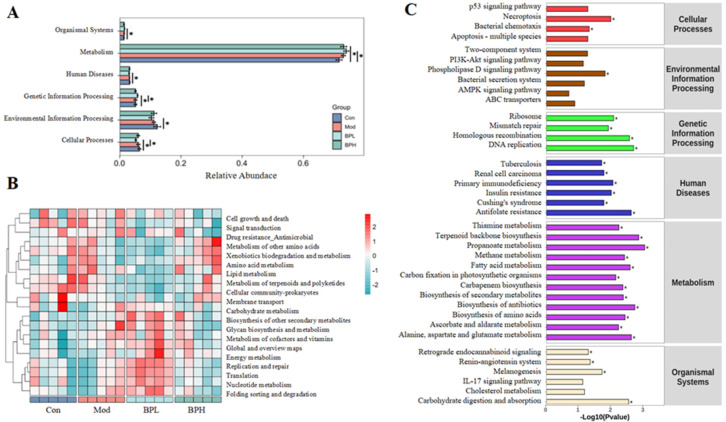
Effect of BP on gut microbial functions. (**A**) KEGG of microbial (level 1). (**B**) Heatmap of KEGG pathways (level 2). (**C**) KEGG pathways (level 3). * *p* < 0.05.

The KEGG database can be divided into three hierarchical levels, and comparing the database with the microbiological results can reveal metabolic pathways at different levels significantly. In Figure 9A, the results of level 1 demonstrated that the broad categories of the biological processes of the microorganisms within the metabolic pathways have changed significantly after the BP intervention. Before the intervention, genes were mainly enriched in cellular processes and environmental information processing, while after the intervention, gene enrichment was mostly focused on metabolism, genetic information processing, and organismal systems. A heatmap of KEGG pathways showed that the low-dose BP significantly regulated 10 pathways at level 2 (specific pathways within broad categories), such as energy metabolism, nucleotide metabolism, glycan biosynthesis, and the metabolism and biosynthesis of other secondary metabolites (Figure 9B). The high-dose BP could improve the metabolism of amino acid and membrane transport compared with the Mod group. Further analysis found (Figure 9C) that BP affected multiple signaling pathways and was associated with the emergence and progression of various diseases, including tuberculosis, primary immunodeficiency, and insulin resistance. The following metabolic pathways were significantly enhanced after the intervention of BP: carbohydrate digestion and absorption, alanine, aspartate and glutamate metabolism, cholesterol metabolism, fatty acid metabolism, and the biosynthesis of secondary metabolites. It was indicated that BP was believed to serve as an energy source for the gut microbiota, enhancing the activity of metabolic enzymes and improving the synthesis and metabolic capacity of nutrients within the body. These findings indicated the potentially positive metabolic remodeling in the gut microbiota affected by BP during the progression of lung cancer.

## 4. Discussion

BP is a typical non-starch polysaccharide with a molecular weight of 70.4 kDa, mainly containing glucose, galactose, rhamnose, and mannose and galacturonic acid [11]. Polysaccharides are natural bioactive substances found in many plants that cannot be digested by gastrointestinal enzymes but can be fermented by bacteria. They act as prebiotics by altering the composition of the gut microbiota, strengthening epithelial integrity, regulating microbial metabolism, and activating or inhibiting innate immune cells [29,30]. Healthy microbial communities can improve the function of the epithelial barrier, mitigate inflammation, and prevent related disorders as well as alleviate associated symptoms [16,31]. Strategies for modulating the gut microbiota have been described to benefit cancer patients and serve as innovative prognostic biomarkers for treatment response. Therefore, the current study was dedicated to determining the effect of BP on the composition and function of intestinal microbiota with lung cancer through the intervention.

In the experiments, we found that BP could reduce tumor weight, suppress Ki67 expression, and promote tumor cell apoptosis. Angiogenesis plays an important role in the occurrence and development of tumors. TGF-β can achieve immune suppression by inhibiting the proliferation and function of T cells; TGF-β and PDGF-BB can also induce the secretion of VEGF, promoting the formation of vascular and tumor interstitial fibers [32,33,34]. The ingestion of BP could reduce levels of vascular growth factors and effectively inhibit the abnormal proliferation of tumor cells. Chronic inflammation is closely associated with the development of cancer, and natural polysaccharides indirectly inhibit tumor growth primarily by enhancing the immune system [35]. The levels of inflammatory factors such as IL-1β, IL-6, TNF-α, and CRP in the serum of the lung cancer mice increased, promoting the activation of inflammatory cells and stimulating the release of inflammatory mediators, leading to an increased risk of systemic inflammatory response. However, BP intervention corrected the tumor-induced inflammatory trends mentioned above, especially TNF-α, which could disrupt the barrier of the intestinal epithelium and induce the synthesis of pro-inflammatory cytokines by intestinal epithelium and macrophages, which had the pathogenicity of the intestine [36].

Currently, growing evidence suggests that the etiology of lung cancer is associated with anomalies of the gut–lung axis. Pathological conditions such as cancer, trauma, stress, and inflammation damage the intestinal barrier to varying degrees, aggravating the primary disease [16,37]. Inflammatory cytokines are significantly elevated in intestinal inflammatory diseases, and the overexpression of IL-1β and TNF-α can induce increased permeability of the tight junction barrier of the intestinal epithelium, activating intestinal immunity [24]. Although there was no significant difference in the levels of IL-1β, IL-6, and TNF-α mRNA between the Con group and the Mod group in this finding, the inhibition of these markers by the BP treatment group suggested that BP has a potential regulatory effect on gut inflammation. In addition, IFN-λ has antiviral effects in the gastrointestinal tract and can promote the regeneration of intestinal epithelial cells after injury, maintaining the dynamic balance of the intestinal mucosal barrier [25,38]. J. Ouaknine et al. revealed the role of intestinal barrier markers in immune suppression points and prognosis in NSCLC patients through the evaluation of intestinal barrier markers in plasma [39]. Meanwhile, the combination therapy of ginseng polysaccharides could lessen the infiltration of inflammatory cells in the colon [4]. Our experiments revealed that LLC tumor-bearing mice suffered from severe enteritis, and the protein levels of Occludin and ZO-1 in colon tissue were significantly reduced, leading to impaired intestinal barrier function.

Natural polysaccharides can mitigate intestinal damage by suppressing pro-inflammatory mediators, enhancing the intestinal barrier, and modifying the gut microbiota composition [30]. We observed that there was no substantial and consistent change in the diversity of microbial communities before and after intervention in the groups. In fact, BP intervention may even lead to lower α-diversity values of the gut microbiota in lung cancer. However, the purpose of supplementing BP is to allow certain gut microbial populations to proliferate over others in order to promote the production of beneficial metabolic products [40]. Furthermore, a reduced ratio of F/B in the Mod group may lead to a decrease in circulating SCFAs and thus affect inflammation and host systemic immunity. At the genus level, *Blautia* demonstrated a significant rise in the tumor-bearing mice after the intervention of BP. A significant amount of research has recently focused on the probiotic properties of *Blautia*, including biotransformation capabilities and its capacity to regulate host health and relieve metabolic syndrome [41]. Members of *Bifidobacterium* possess diverse probiotic properties, contribute to anticancer effects, and reduce the inflammatory effects of TNF-⍺ and lipopolysaccharides [42]. Research has also found that *Bifidobacterium* and *Olsenella* significantly enhanced the efficacy of immune checkpoint inhibitors in cancer-bearing mice [43]. Members of the Lactobacillus interact with the mucosal immune system, inducing epithelial cells to produce antimicrobial peptides and promoting mucosal homeostasis [44]. The abundance of *Limosilactobacillus* and *Ligilactobacillus*, which have antimicrobial and immune as well as intestinal barrier-enhancing abilities, were inhibited in the lung cancer-bearing mice. *Faecalibaculum* produced SCFAs that contributed to regulating tumor cell proliferation in mice and human settings without affecting adaptive immune cells [45]. Therefore, BP could promote the proliferation of microbial communities associated with gut immunity and tumor suppression while preventing the growth of certain pathogenic bacteria.

Previous studies have shown that acetic acid, butanoic acid, and isovaleric acid were the primary metabolites of turnip polysaccharides [15]. We also found that all the SCFAs, including acetic acid, propanoic acid, butanoic acid, isovaleric acid, and valeric acid, increased after the intervention of high-dose BP. The primary SCFAs, including acetate, propionate, and butyrate, have a crucial role in regulating immunity, cell apoptosis, inflammation, and lipid metabolism. Specifically, butyrate is important in preventing the onset and development of cancer by regulating cell division, apoptosis, and differentiation [46]. The intake of BP can increase the relative abundance of butyrate-producing bacteria including *Dubosiella* [47] and *Erysipelotrichaceae* [48], presenting a protective effect against immune system dysfunction. According to the KEGG, BP could promote carbohydrate digestion and absorption; alanine, aspartate, and glutamate metabolism; cholesterol metabolism; and fatty acid metabolism. The alterations in amino acids, fatty acids, and lipids, along with their associated metabolic pathways, are related to the immunotherapeutic efficacy against NSCLC [49]. Hence, the protective effects of BP against lung cancer were attributed to reversing alterations in the gut microbiota.

In conclusion, we confirmed the impact of BP on lung cancer mice by inhibiting inflammatory response, maintaining intestinal homeostasis, and regulating intestinal metabolism. Furthermore, a diet rich in fibers can prevent tumor development in a microbiota-dependent manner [50]. These suggest that BP represents a source of prebiotics and has the potential to intervene in lung cancer progression by targeting the microbiota. Certainly, utilizing BP as a feasible strategy to enhance drug absorption and efficacy requires well-designed studies with larger sample sizes and extended clinical trials.

## 5. Conclusions

In this study, the in vivo investigations in LLC tumor-bearing mice revealed that BP could inhibit tumor growth and reduce the serum levels of inflammatory factors and growth factors. Furthermore, BP intervention contributes to the suppression of intestinal inflammation, the preservation of intestinal barrier completeness, and the enrichment of beneficial microbial populations, including *Blautia* and *Bifidobacterium*. The increase in *Blautia*, *Dubosiella*, and *Faecalibaculum* by BP supplement contributed to the production of SCFAs, which played a protective role against metabolic diseases and tumors. In addition, the intake of BP may benefit specific metabolic functions of the gut microbiota, such as amino acid metabolism, cholesterol metabolism, and fatty acid metabolism. In summary, this study illuminates the multifaceted impact of BP intervention in the context of lung cancer and gut microbiota modulation. These findings provide evidence for the further exploration of BP as a potential tool for disease prevention and as an adjuvant in lung cancer treatment strategies.

## Figures and Tables

**Table 1:** Primer sequence of RT-qPCR.

	Upstream Primer (5′-3′)	Downstream Primer (5′-3′)
β-actin	CATTGCTGACAGGATGCAGAAGG	TGCTGGAAGGTGGACAGTGAGG
IL-1β	GCAACTGTTCCTGAACTCAACT	ATCTTTTGGGGTCCGTCAACT
IL-6	GATGAACCATCTCCGTTGGC	CCCAATTATGAATCGGGAGTGC
TNF-α	AAGGACCTGGTACATGAACTGG	CGGACCATAGAGAGTGGAAAGG
IFN-γ	GTGGCAGCTACCTGTGTCTT	CTCTGCTTGTGAGGTGCTGA
ZO-1	GCTTTAGCGAACAGAAGGAGC	TTCATTTTTCCGAGACTTCACCA
Occludin	CTGGATCTATGTACGGCTCACA	TCCACGTAGAGACCAGTACCT

## Data Availability

The raw data supporting the conclusions of this article will be made available by the authors on request.
